# Significance of pleural effusion at diagnosis in pediatric Hodgkin lymphoma: a report from Children’s Oncology Group protocol AHOD0031

**DOI:** 10.1007/s00247-018-4197-6

**Published:** 2018-07-16

**Authors:** Kathleen M. McCarten, Monika L. Metzger, Richard A. Drachtman, Qinglin Pei, Debra L. Friedman, Cindy L. Schwartz, Kara M. Kelly

**Affiliations:** 1 Diagnostic Imaging, Warren Alpert Medical School, Brown University, 222 Richmond St., Providence, RI 02903 USA; 2 Pediatric Radiology, IROC Rhode Island/Quality Assurance Review Center, Lincoln, RI USA; 3 Department of Oncology and Global Pediatric Medicine, St. Jude Children’s Research Hospital, Memphis, TN USA; 4 Department of Pediatric Hematology-Oncology, Rutgers Cancer Institute of New Jersey, New Brunswick, NJ USA; 5 Department of Biostatistics, COG Data Center, University of Florida, Gainesville, FL USA; 6 Department of Pediatrics, Vanderbilt Ingram Cancer Center, Nashville, TN USA; 7 Department of Pediatrics, Children’s Hospital of Wisconsin, Milwaukee, WI USA; 8 Department of Pediatrics, Roswell Park Cancer Institute, Buffalo, NY USA

**Keywords:** Children, Computed tomography, Hodgkin lymphoma, Pleural effusion, Positron emission tomography, Prognostic features, Thorax

## Abstract

**Background:**

Pleural effusion at presentation in Hodgkin lymphoma has been associated with inferior outcome but has not been systematically evaluated.

**Objective:**

To determine whether pleural effusion at presentation in children with Hodgkin lymphoma is a primary indicator of poor prognosis or secondary to associated factors.

**Materials and methods:**

Children’s Oncology Group (COG) AHOD0031, a randomized, response-based, centrally reviewed protocol, enrolled 1,712 eligible patients <22 years of age with initial presentation of intermediate risk, biopsy-proven Hodgkin lymphoma; 1,423 had available imaging for retrospective review. We coded effusions as fluid-only or with associated pleural nodule or adjacent lung or bone involvement and correlated this with disease stage, tumor response, large mediastinal adenopathy, and mass effect on the superior vena cava (SVC) and left innominate vein. We recorded change in size and character of effusions post-chemotherapy.

**Results:**

Pleural effusions were present in 217, with 204 having fluid-only and 13 having associated solid components. Patients with effusions were more likely to have large mediastinal adenopathy (*P*<0.0001), be slow early responders (*P*<0.0001) and have higher relapse rate (*P*<0.0001). Vascular compression was not significantly correlated with pleural effusion. Of 121 patients with adequate [F-18]2-fluoro-2-deoxyglucose (FDG) positron emission tomography (PET)/CT imaging, no FDG PET avidity was seen in any pleural effusion but was present in solid components. The side of the pleural effusion in those with moderate or large effusions was highly associated with the side of large mediastinal adenopathy (*P*<0.0001). Statistical analysis indicates that pleural effusion is an independent risk factor for poorer response and relapse.

**Conclusion:**

Pleural effusion in Hodgkin lymphoma is an important independent poor prognostic indicator for response and relapse.

## Introduction

Pleural effusion has been reported in 7–30% of patients with Hodgkin lymphoma [1–4]. It is more common in adults than children at both presentation and relapse, and its presence has been considered a poor prognostic factor in limited studies [2, 3]. An association between pleural effusion and large mediastinal adenopathy has long been recognized [3, 5–8], where large mediastinal adenopathy is a mediastinal mass with transverse diameter on a posteroanterior (PA) upright chest radiograph ≥33% of the diameter of the thorax at the level of the diaphragm. Most pleural effusions in Hodgkin lymphoma are chylous and devoid of malignant cells [6, 8–10], suggesting that invasion of disease into the pleural space is not usually the primary etiology. It has been suggested that pleural effusions arise primarily from thoracic duct compression or rupture [3, 11, 12], compression of the intrinsic lymphatic channels of the lung and thorax, compression of major venous channels [11–14] or increased capillary permeability from inflammation or overt endothelial damage [13, 15]. Non-traumatic disruption of the thoracic duct is considered less likely because thoracic duct disruptions from other causes rarely resolve without intervention [16].

Knowledge of the lymphatic and vascular structures of the thorax and their interconnections is essential to understand possible anatomical etiologies for pleural effusions. The intrinsic lymphatic drainage of the lung consists of subpleural channels that extend through the interstitium and along perivascular structures, then draining into hilar and subcarinal nodes and into the mediastinum. Mediastinal, subcarinal or hilar adenopathy can prevent normal drainage, producing elevated hydrostatic pressure and causing transudation of fluid [3, 6–8, 13, 14]. The thoracic duct, the major egress of lymphatics from below the diaphragm, drains into the vascular system where the left jugular and left subclavian veins join to form the left innominate vein. Its drainage can be impeded by direct compression by a large mediastinal mass or secondarily by vascular compromise of the left innominate vein central to the thoracic duct’s insertion into the vein, preventing normal drainage, with either mechanism producing elevated hydrostatic pressure and transudation [6–8, 11, 17]. Compromise to the lumen of the SVC by adenopathy can cause diminished cardiac venous return, potentially resulting in SVC syndrome. Three small published series of SVC syndrome in children with malignancies verified Hodgkin lymphoma in 2 of 10, 2 of 24, and 2 of 11 cases, respectively [18–20], but overall incidence has not been reported and its presence in this protocol was not captured.

Routine imaging in the pre-CT era could not differentiate potential etiologies of pleural effusion. With contemporary imaging, it is now appropriate to re-evaluate the possible etiology and clinical impact of pleural effusion at time of presentation in newly diagnosed Hodgkin lymphoma and its relationship to clinical and anatomical components of the disease. The large number of patients treated with the same chemotherapy backbone made this an ideal study for evaluating the presence of pleural effusion, its characteristics and associated factors, as well as its prognostic significance. In addition, contrast-enhanced CT makes it possible to evaluate the potential for development of SVC syndrome.

## Materials and methods

Children’s Oncology Group (COG) AHOD0031, an intermediate-risk clinical trial for patients with newly diagnosed Hodgkin lymphoma, was designed to evaluate whether response-based therapy would support the goal of maintaining excellent cure rates while avoiding treatment-associated risks that compromise long-term health [21]. To this end, subsequent therapy was adapted based on early radiographic response to chemotherapy after two cycles of ABVE-PC (doxorubicin, bleomycin, vincristine, etoposide, prednisone and cyclophosphamide). This protocol was approved by the National Cancer Institute and participating institutional review boards and was open for enrollment from September 2002 to July 2009. All diagnoses were biopsy proven. Staging was based on the Ann Arbor staging classification depending on sites of involvement and classified as absence (A) or presence (B) of systemic symptoms such as unexplained fevers, drenching night sweats and unintentional weight loss. This intermediate-risk protocol included stages IB, IAE (E=extranodal extension of disease), IIB, IIAE, IIIA, IVA, with or without bulk disease, and IA and IIA with bulk disease. Bulk disease was defined as either a large mediastinal adenopathy or an extramediastinal nodal mass ≥6 cm in transverse diameter on axial CT (e.g., cervical, retroperitoneal, axillary). Low-risk stages IA and IIA without bulk were excluded, as were high-risk stages IIIB and IVB.

**Fig. 1:**
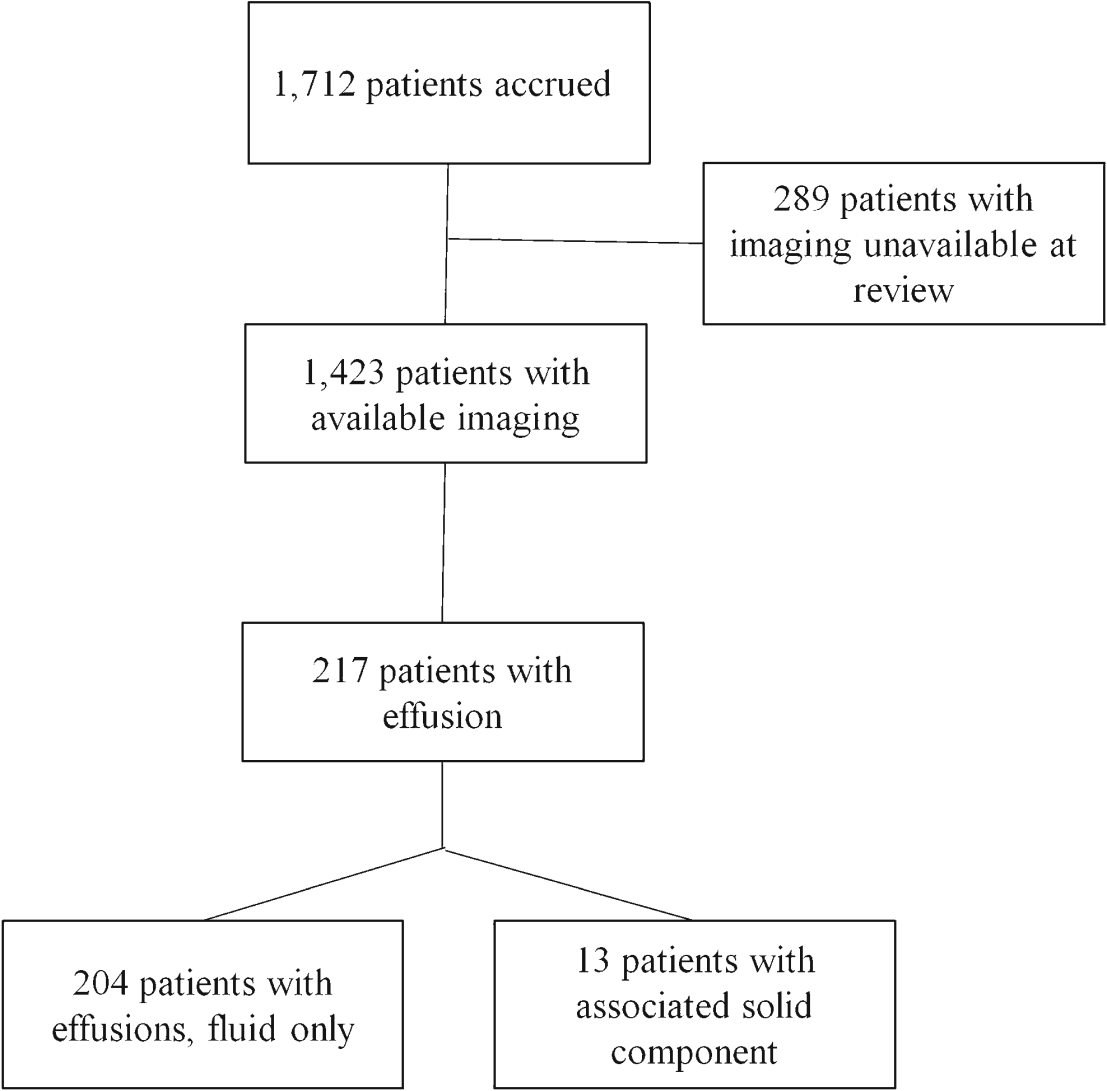
Flow diagram of patient selection

At study entry the required imaging included upright PA and lateral chest radiographs, contrast-enhanced CT of neck, chest, abdomen and pelvis, and either [F-18]2-fluoro-2-deoxyglucose (FDG) positron emission tomography (PET), FDG PET/CT, or gallium scan where PET was not yet available. Repeat contrast-enhanced CT of neck, chest, abdomen and pelvis was obtained after two treatment cycles and at the conclusion of chemotherapy, i.e. after four cycles of chemotherapy. FDG PET or gallium was obtained after two cycles in all patients; it was obtained at the conclusion of chemotherapy only in those who were positive after two cycles. All imaging was compliant with the Health Insurance Portability and Accountability Act. Of the 1,712 patients enrolled in this protocol, 1,423 had available imaging in the computer system to qualify and quantitate pleural effusion and are included in this analysis; images sent on hard copy film or non-uploadable discs were excluded from this analysis (Fig. 1). Data were sent to IROC RI (Imaging and Radiation Oncology Core Rhode Island), formerly QARC (Quality Assurance Review Center) by disc, Digital Imaging and Communications in Medicine (DICOM), or direct image transfer where it was de-identified, stored and available for central review. This protocol was initiated prior to the ALARA (as low as reasonably achievable) principle of imaging being widely utilized, but all imaging factors were provided by the institutions and were in compliance with the protocol criteria and in the acceptable range for the pediatric population for the period of time during which they were acquired.

Real-time rapid central review of imaging after two cycles of chemotherapy was obtained at IROC RI to determine early response and thus tailor subsequent therapy. Patients were categorized as rapid early responders (≥60% decrease in mass size) or slow early responders (<60% decrease). Slow early responder patients went on to routine completion of therapy. For rapid early responder patients, rapid central review was again obtained after four cycles of chemotherapy and patients categorized as complete responder (≥80% reduction in mass size and nuclear medicine negative, i.e. PET avidity below mediastinal blood pool or disappearance of any gallium activity) or <complete responder. This protocol occurred prior to the development of the Deauville score for assessing FDG PET response; a negative assessment in this protocol was the equivalent of a Deauville 1 or 2 score. Therapy management was based on early and complete response categorization [21]. Statistical inference (chi-square test on categorical variables and analysis of variance [ANOVA] test on continuous variables) was performed to compare the following demographics and clinical conditions between patients with and without pleural effusions: age, gender, stage, histology, large mediastinal adenopathy and response to chemotherapy. A Cox regression was performed to test the effect of pleural effusion on time to relapse by considering the following covariates: age, gender, stage, histology, large mediastinal adenopathy, response and B symptoms, among which gender, histology and B symptoms were removed from the multivariate regression because of large *P*-values (>0.15). In addition, we performed a logistic regression analysis of response rate (slow early responders vs. rapid early responders) considering the following covariates; age, gender, stage, histology, large mediastinal adenopathy and B symptoms. For all statistical tests, we will consider the comparisons results in significance difference if the *P*-value is <0.05.

**Fig. 2:**
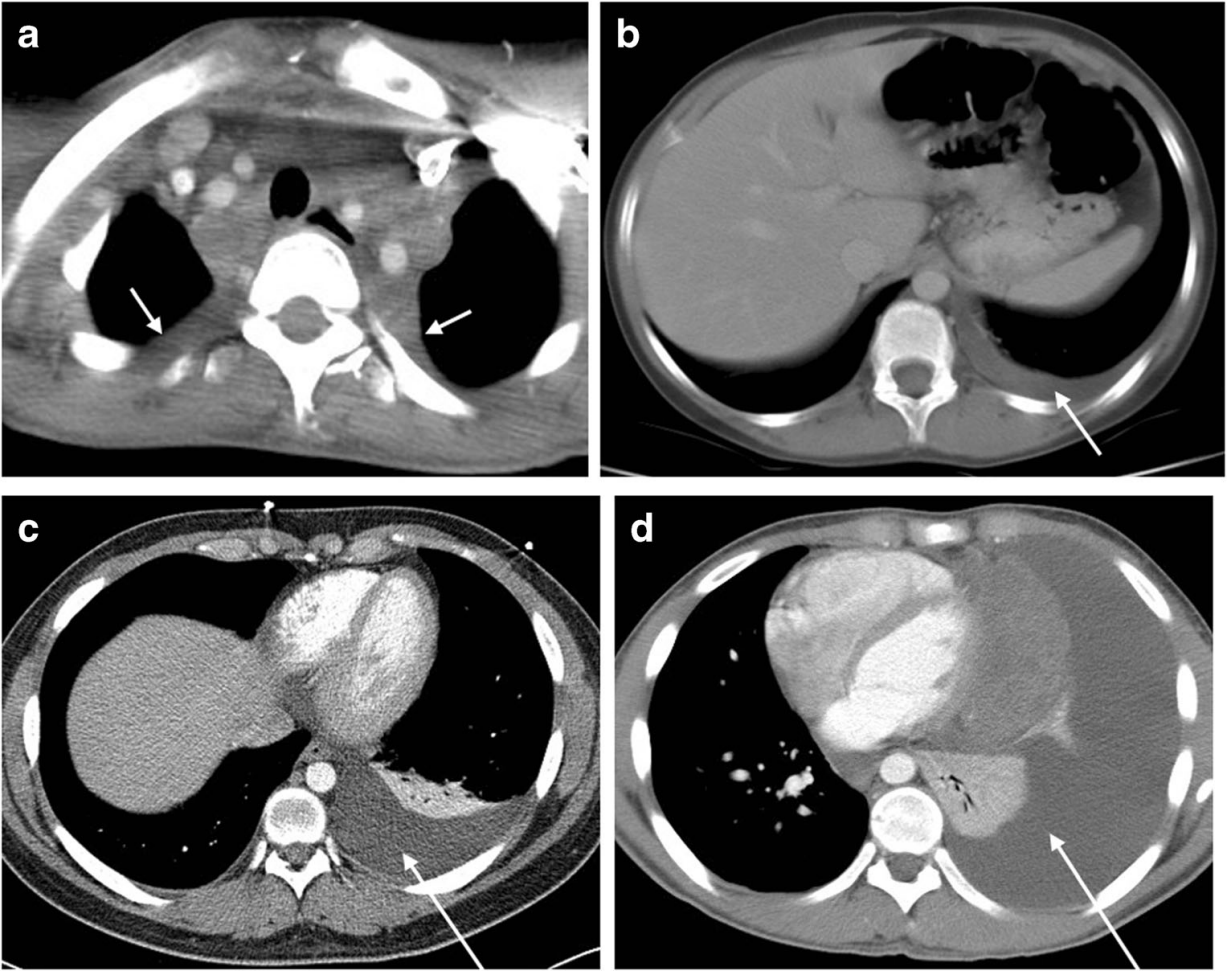
Effusion categories. **a** Trace effusion (*arrows*), with at least 1 cm of fluid inferiorly or 1 cm at the apices, as seen here on axial CT image in a 15-year-old girl. The majority of trace effusions were at the apex (129 of 217). This is a distinct finding associated with large mediastinal masses and might reflect transudation from small focal lymphatic channels. **b** Small effusion (*arrow*), seen here in an 11-year-old girl on axial CT image, is any effusion 1–3 cm in size. **c** Moderate effusion (*arrow*) is any effusion >3 cm in size that reached to mid-thoracic level, as seen in a 16-year-old girl on axial CT image. **d** Large effusion (*arrow*), any effusion that extends from the lung base to the apex and displaces heart and mediastinum toward the opposite side, is seen here on axial CT image in a 14-year-old boy

The 1,423 patients included in this retrospective analysis were all rapid early responder and slow early responder patients with complete CT imaging in the computer system with all evaluations being performed pre-chemotherapy, post two cycles and at end of chemotherapy (post four cycles) prior to any involved field radiation therapy. In this retrospective analysis, we developed criteria for presence, extent, laterality and composition of the pleural effusion. We quantified effusions as unilateral or bilateral and as follows: trace, or at least 1 cm of fluid at the apex or 1 cm or less inferiorly in the thorax, measured from the pleural effusion interface with the lung to the chest wall; small, or any effusion 1–3 cm from lung edge to chest wall; moderate, or any effusion >3 cm from lung margin to chest wall that reached to the mid-thoracic level; and large, or any effusion that extended from the lung base to, or near, the apex and displaced heart and mediastinum toward the opposite side (Fig. 2). Additionally, effusions were classified as either pleural-fluid-only or those exhibiting any solid component such as pleural nodules or adjacent lung or bone lesions. Any FDG PET or gallium activity within the pleural space was documented to determine the presence of a focal pleural-related lesion. Data regarding the therapeutic and diagnostic use of thoracentesis was not available because this was not required by the protocol.

Configuration of large mediastinal adenopathy as being midline or obviously asymmetrically positioned within the thorax toward right or left was recorded and correlated with sidedness of effusions in those with moderate or large effusions. In patients with large mediastinal adenopathy and pleural effusions plus adequate intravenous contrast bolus at all time periods and without the presence of an obscuring central venous catheter, we evaluated the appearance of the SVC and left innominate vein and recorded whether it was of normal patency, slightly compressed (<50% decrease in transverse diameter), compressed (>50% decreased diameter) or obliterated. We also documented degree of restoration of the vascular caliber after chemotherapy. To determine whether compression of SVC or left innominate vein alone was the etiology of pleural effusion, a random selection of 208 protocol patients without pleural effusion but with large mediastinal adenopathy and with a similar distribution of disease stage and adequate intravenous contrast bolus were similarly evaluated for SVC and left innominate compression/obliteration pre- and post-chemotherapy. We recorded resolution of pleural effusions and change of any solid features after chemotherapy and prior to any radiation therapy.

## Results

**Fig. 3:**
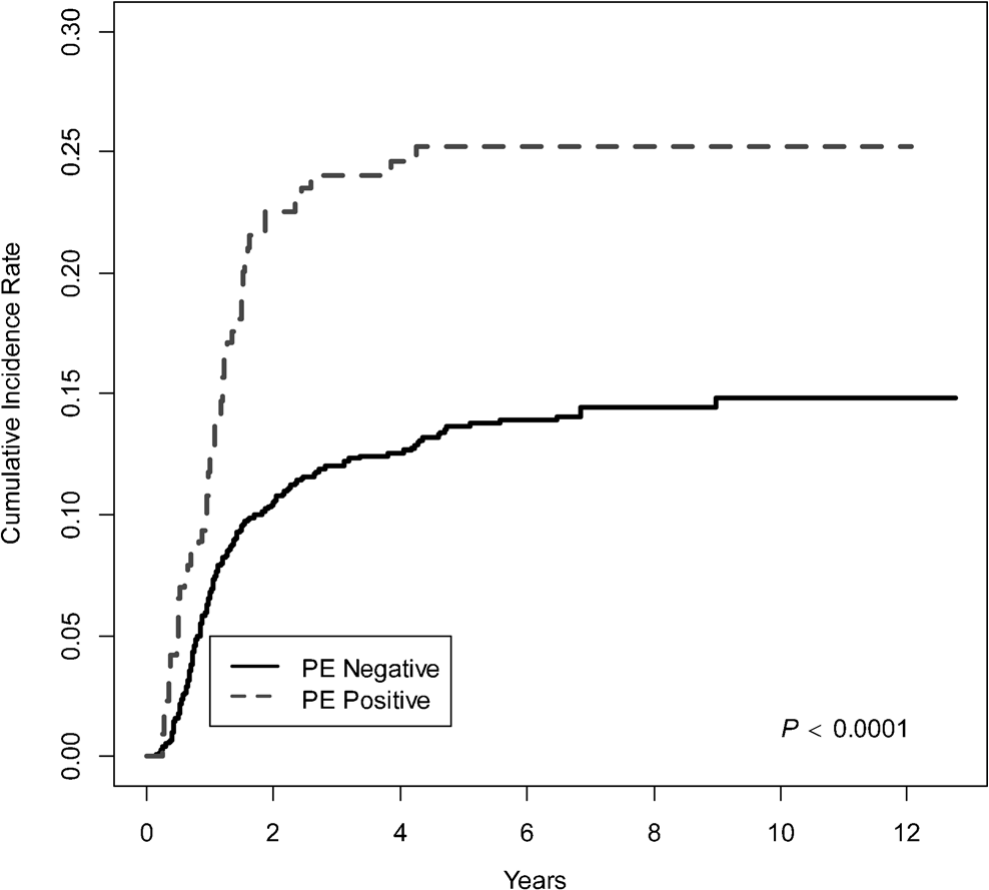
The cumulative incidence rate of relapse in patients who are pleural effusion positive (*dotted line*) vs. pleural effusion negative (*solid line*) is statistically significant (*P*<0.0001; *P*<0.05 considered statistically significant). This demonstrates that the vast majority of relapses in patients with pleural effusion (*dotted line*) occurred within the first 2 years after completion of therapy, with only small numbers relapsing in the next 2 years, and that they occurred at a much more rapid rate than relapses in the pleural-effusion-negative group (*solid line*). *PE* pleural effusion

There were differences in demographics and disease presentation among those with pleural effusion (*n*=217) and those without pleural effusion (*n*=1,206; Table 1). By direct comparison those with pleural effusion were older (15.5 vs. 14.4, *P*<0.0001), more likely to have large mediastinal adenopathy (73% vs. 33%, *P*<0.0001), more likely to be female (54% vs. 46%, *P*=0.02), most likely have stage II disease (70% vs. 57%, *P*=0.0006), more likely to have nodular sclerosing histology (90% vs. 79%, *P*<0.0001), and more likely to have B symptoms (39% vs. 19%, *P*<0.0001). In considering response to chemotherapy, a significantly greater number of patients with pleural effusion were slow early responders compared to those with no pleural effusion (34% vs. 16%, *P*<0.0001; Table 1). The comparison of cumulative incidence rates of relapse over time showed that there were significant differences between the pleural effusion group and the remainder of patients. (Fig. 3; *P*<0.0001). Of those with pleural effusion, 26 (12%) relapsed in the first year after ending therapy, 21(10%) in the second year, and only an additional 5 (2%) relapsed in the subsequent 3 years, as compared to the remainder of the cohort, where 7% relapsed in the first year and 5% the second year. From Fig. 3 it can be seen that this represented a greater number and more rapid rise than the control group.

**Table 1:** Demographic comparison for patients with pleural effusion present (PE+) and without pleural effusion (PE-)

	All	PE-	PE+	*P-*value^a^
*n*	1,423	1,206	217	
Age (mean ± se)	14.6±0.087	14.4±0.098	15.5±0.17	**<0.0001**
Gender				
Male	756	657 (54%)	99 (46%)	**0.02**
Female	667	549 (46%)	118 (54%)	
Stage				
I	82	79 (7%)	3 (1%)	**0.0006**
II	838	686 (57%)	152 (70%)	
III	297	262 (22%)	35 (16%)	
IV	206	179 (15%)	27 (12%)	
Histology (predominant)				
Lymphocyte	82	81 (7%)	1 (0.5%)	**<0.0001**
Lymphocyte depleted	3	2 (0.2%)	1 (0.5%)	
Nodular sclerosing	1,154	958 (79%)	196 (90%)	
Mixed cellularity	122	115 (10%)	7 (3%)	
Unknown	62	50 (4%)	12 (6%)	
LMA				
No	814	766 (64%)	48 (22%)	**<0.0001**
Yes	554	395 (33%)	159 (73%)	
Unknown	55	45 (4%)	10 (5%)	
Response				
RER	1,135	995 (83%)	140 (65%)	**<0.0001**
SER	264	190 (16%)	74 (34%)	
Unknown	24	21 (2%)	3 (1%)	

^a^Chi-square test performed on categorical variables and analysis of variance (ANOVA) test on continuous variables. If the *P*-values of the comparison are smaller than 0.05, it is considered that there are significant differences between the two groups. Any significant findings will be represented with *P*-values in bold font

*LMA* large mediastinal adenopathy, *RER* rapid early responders, *se* standard error, *SER* slow early responders

In the Cox regression analysis including age, gender, stage, histology, large mediastinal adenopathy, response to therapy and B symptom as co-variates, pleural effusion remained an independent risk factor for relapse. Also, the logistic regression indicates that pleural effusion played a significant role in terms of response rate (slow early responders vs. rapid early responders, *P*=0.0003), while the following co-variates were also considered: age, gender, stage, histology, large mediastinal adenopathy and B symptoms.

**Fig. 4:**
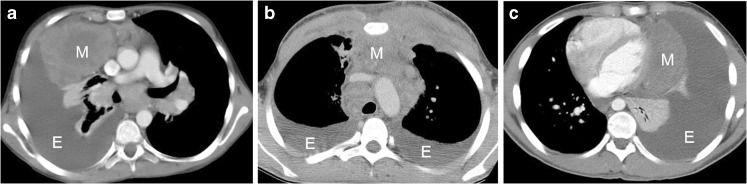
Axial CT images of mediastinal masses and associated pleural effusions. Most patients with eccentrically positioned masses toward one hemithorax or the other and moderate or large effusions demonstrated a much higher likelihood of only ipsilateral effusions (**a, c**), while a more centrally positioned large mediastinal adenopathy presents with bilateral effusions (**b**). **a** A 16-year-old girl; (**b**) another 16-year-old girl; (**c**) a 7-year-old boy. *E* effusion, *M* mass

Of the 1,423 patients evaluated, 217 demonstrated pleural effusion at diagnosis, the characteristics of which relative to laterality, size and character are listed in Table 2. Forty-nine patients had moderate and large effusions (including 4 bilateral=53 effusions) and all had large mediastinal adenopathy. Of those with a predominantly right-side mediastinal mass, 11 of 12 had only a right-side effusion, and of those with a left-side mass, 17 of 18 had only a left-side effusion. This indicates that the side of the large mediastinal adenopathy is significantly correlated with the side of the pleural effusion (*P*<0.0001; Table 3; Fig. 4).

**Table 2:** Characteristics of 286 pleural effusions in 217 affected patients

Characteristic	*n* (%)
Laterality	
Unilateral	148 (68)
Bilateral	69 (32)
Side of effusion*	
Right	131 (46)
Left	155 (54)
Size of effusion*	
Trace	159 (56)
Small	74 (26)
Moderate	39 (14)
Large	14 (5)
Character of effusions	
Fluid alone	204 pts. (273 effusions)
Solid components	13 pts. (13 effusions)

*Calculated for a total of 286 effusions in 217 patients when each side was considered separately

*pts* patients

**Table 3:** Relationship between side of pleural effusion with side of large mediastinal adenopathy (LMA) for patients with moderate and large pleural effusions (relationship is statistically significant. The significance level of *P*-value is 0.05)

		LMA location		
		Left	Midline	Right
Effusion location	Bilateral	1	3	0
	Left	17	8	1
	Right	0	8	11

*P*-value<0.0001

The size of the effusion had no significant relationship to therapy response. Those with moderate and large effusions had virtually the identical incidence of slow early response as did the entire cohort of those with pleural effusions. Of the 286 effusions in the 217 patients, 273 were fluid-only and 13 had an associated solid component.

**Fig. 5:**
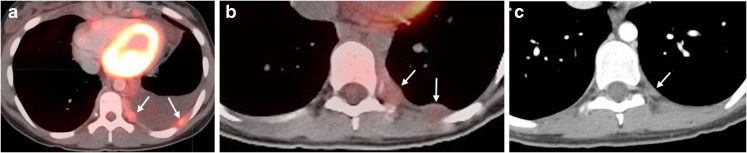
Example of complex effusion. **a** Pre-study PET/CT exam in a 14-year-old girl demonstrates FDG PET avidity in pleural nodules with associated moderate effusion. **b, c** Imaging in a 16-year-old girl with two nodules seen initially on an axial PET/CT image (**b**), with one small residual post-therapy nodule as seen on an axial CT image (**c**)**.** *FDG* [F-18]2-fluoro-2-deoxyglucose, *PET/CT* positron emission tomography/computed tomography

Among 121 patients with available FDG PET imaging, all effusions were FDG PET non-avid. The associated solid components included pleural nodules alone (*n*=9), adjacent peripheral lung nodules (*n*=2), adjacent rib lesion (*n*=1) and both lung and pleural nodules identified (*n*=1). Eight of the nine patients with pleural nodules alone had trace effusion while one had a moderate effusion (Fig. 5).

Ten of the 13 patients with associated solid components were evaluated by FDG PET and three were evaluated by gallium. Of the 10 who were evaluated by FDG PET, all nodules and adjacent involved lung or bone were PET-avid. Those who had gallium scans showed no gallium positivity in any lesion.

**Fig. 6:**
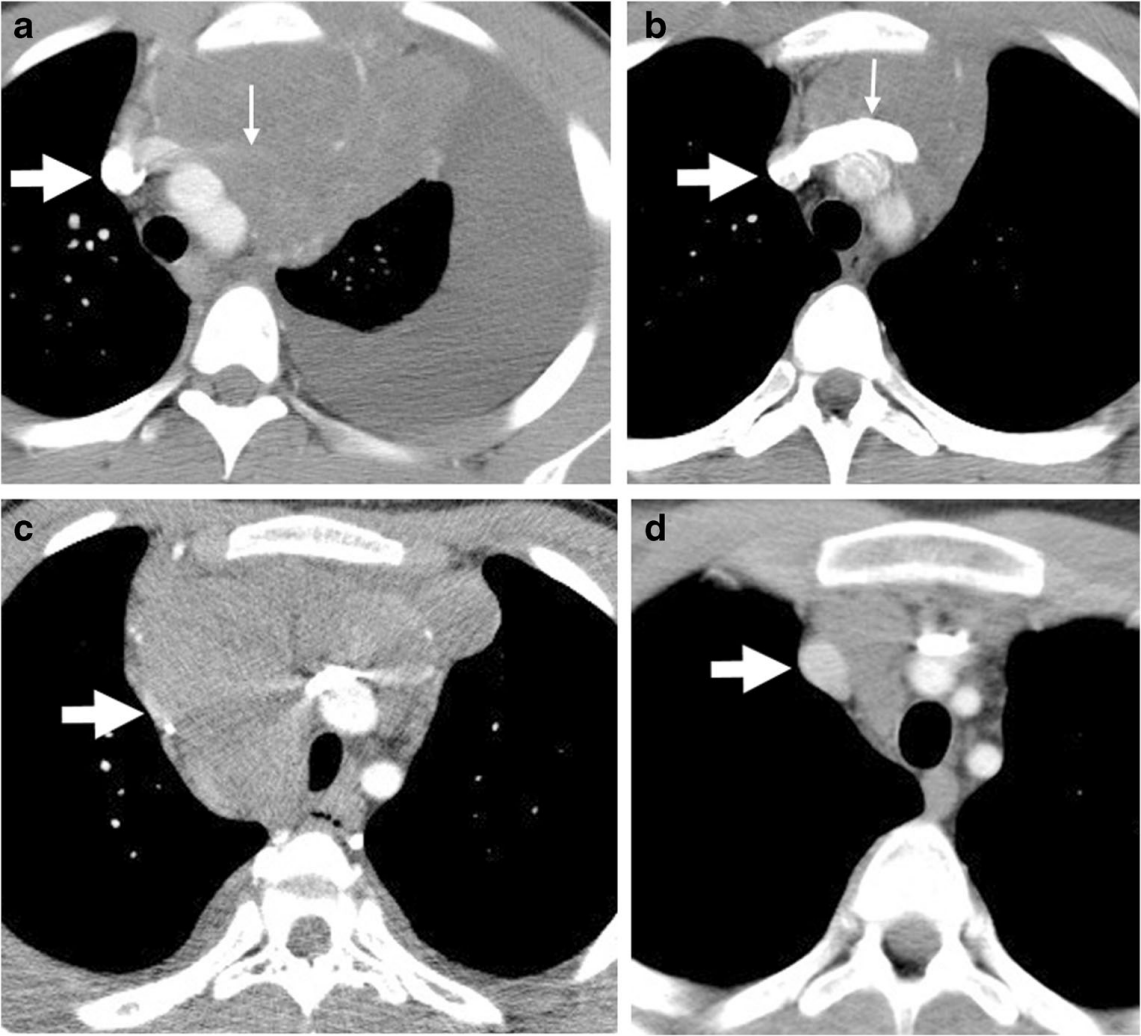
Vascular compression by mediastinal mass. **a** Pre-study axial CT image in a 7-year-old boy shows the contrast-enhanced left innominate vein is obliterated (*thin arrow*); (**b**) post-therapy, axial CT shows the caliber of the left innominate vein has been reconstituted. The SVC in both images of this boy (*broad arrow*) is patent. **c, d** Axial CT images in a 14-year-old boy show almost total compression of the SVC laterally on the pre-therapy study (**c,** *arrow*), which returns to normal caliber as seen in (**d**) post-therapy. *SVC* superior vena cava

Of the 217 patients with pleural effusion 188 (87%) had adequate intravenous contrast bolus on all scans for evaluation of vascular patency and no occluding central catheter, compared to 183 (88%) of the 208 random controls with large mediastinal adenopathy but no effusion and similar disease stage. When the SVC was evaluated for patency in both groups, there was no significant difference in extent of patency between the effusion group and those without effusion (Table 4). The same held true for the left innominate vein patency. An imaging pattern was seen, however, in the difference in the effect of adenopathy on the SVC and innominate vein (Fig. 6). Because of the location of the SVC in the right lateral superior mediastinum, it was more often simply displaced laterally (62%) rather than being circumferentially compressed (38%). The left innominate vein, on the other hand, traverses the superior mediastinum from left to right and so is more significantly impacted by adenopathy, particularly large mediastinal adenopathy, such that in 86.7% of cases it was circumferentially surrounded by mass or significantly compressed between mass and aorta and in only 13.3% was there superior displacement only. This difference in location and configuration in the pleural effusion group might account for fewer SVCs (*n*=8) still having some element of compression post chemotherapy completion when compared to the left innominate vein (*n*=30; Table 4).

**Table 4:** Superior vena cava (SVC) and left innominate vein comparison (chi-square test) on contrast-enhanced CT between patients with pleural effusion and a random matched control group without effusion

		Total patients ALL	Patients with pleural effusion	Matched cohort of patients without pleural effusion	*P*-value
*n*		425	217	208	
Stage	I	7	3	4	0.96
	II	298	152	146	
	III	66	35	31	
	IV	54	27	27	
SVC pre-treatment	Normal	93	48	45	0.14
	Slight compression^a^	96	48	48	
	Compression^b^	172	83	41	
	Obliterated^c^			48	
SVC post-treatment	Normal	355	179	176	0.14
	Slight compression^a^	7	2	5	
	Compression^b^	5	5	0	
	Obliterated^c^	2	1	1	
Left innominate pre-treatment	Normal	73	40	33	0.42
	Slight compression^a^	79	88	39	
	Compression^b^	187	88	99	
	Obliterated^c^	31	20	11	
Left innominate post-treatment	Normal	318	157	161	0.06
	Slight compression^a^	21	8	13	
	Compression^b^	19	14	5	
	Obliterated^c^	10	8	2	

^a^Slight compression = <50% narrowing of vessel

^b^Compression = >50% narrowing

^c^Obliterated = total compromise of vessel lumen

The significance level of *P*-value is 0.05

After two cycles of chemotherapy all but 17 effusions had resolved completely. By the end of all chemotherapy all but six effusions had resolved completely; of these six remaining effusions, two were trace, two small, and two moderate. All of the trace effusions occurring at the lung apices resolved. Of the effusions with a solid component, all resolved completely and all nodules decreased to<1 cm by the end of chemotherapy and became FDG-PET-negative, lung lesions resolved completely, and bone lesions became FDG-PET-negative.

## Discussion

This is the largest retrospective study evaluating the significance of pleural effusion at presentation in children and adolescents with newly diagnosed Hodgkin lymphoma. The importance of this study is that multiple aspects of pleural effusion in Hodgkin lymphoma were evaluated, both clinical and anatomical, and the availability of imaging at multiple time points during therapy allowed for excellent characterization of the evolution of effusions with chemotherapy. The incidence of pleural effusions in this study was 15%, which is similar to that reported elsewhere [1–4, 9, 15] and comparable to other studies [1, 4, 6, 17]. Large mediastinal adenopathy (73% in this study) was significantly associated with pleural effusion, which confirms the previously reported association. The presence of pleural effusion was also significantly associated with slow early response (<0.0001). Most important, the presence of pleural effusion represents an independent risk factor for relapse.

Anatomical factors might play some role in formation of pleural effusion but these most likely reflect the impact of the other components of the disease process. The major factor is association with large mediastinal masses, and further supporting it is the correlation of an eccentric position of large mediastinal adenopathy with ipsilateral moderate or large effusions (Fig. 4). Although there is no definite explanation here, the presence of the large number of distinct trace apical effusions (129 of 217) might also relate to large mediastinal adenopathy, possibly secondary to obstruction by the mediastinal mass of smaller lymphatic channels other than the thoracic duct that drain into the vascular system. The total resolution of these trace apical effusions and almost complete resolution of all other effusions with therapy during the progressive shrinkage of adenopathy with chemotherapy also substantiate an anatomical component. The very small number (13 of 217) of solid lesions, i.e. pleural nodules, adjacent lung masses, and adjacent bone lesions, suggests that formation of effusions by more local disease is less common.

This is the first paper with such large numbers of pediatric patients to evaluate mediastinal vascular status in Hodgkin lymphoma secondary to adenopathy, in particular large mediastinal adenopathy. Because there can be significant compression or even obliteration of the lumen of SVC pre-chemotherapy, it is certainly possible that there could be associated clinical SVC syndrome caused by obstruction to normal venous return to the heart. Similarly, compression or obliteration of the left innominate vein could produce decreased venous return to the heart as well as decreased lymphatic return from impaired drainage of the thoracic duct into the left innominate vein. Also demonstrated was that the compromise to the two major vessels had a slightly different mechanism, with the SVC being more frequently compressed by lateral displacement by adenopathy, whereas the left innominate vein was more frequently circumferentially surrounded by adenopathy. Although there was no statistical difference in the appearance of the vascular structures between those with pleural effusion and those without, the finding of persistent vascular compromise in some patients at end therapy could have future clinical implications; for example at end therapy at least two patients with persistent obliteration of the left innominate vein in the pleural effusion group had well-developed collateral circulation on the left side of the thorax, demonstrated on contrast-enhanced CT.

There are a few limitations of this study, including the retrospective nature of the analysis. Second, the protocol reviewed did not include patients in all stages, particularly the highest-risk patients (stages IIIB and IVB), and their inclusion could have strengthened the results. We do not, however, believe that it would have categorically changed the ultimate interpretation of this analysis. Evaluation of pleural effusions in large prospective protocols should be considered to assess whether presence of pleural effusions needs to be taken into account in the risk classification of newly diagnosed patients [22].

## Conclusion

The etiology of pleural effusions in Hodgkin lymphoma is multifactorial. Anatomical factors might play some role in development of pleural effusions, but it is important to note that they are associated with large mediastinal adenopathy, older age, nodular sclerosis histology, stage and, significantly, with slower response to chemotherapy and increased incidence of relapse. This association with poorer response to chemotherapy and increased incidence of relapse independent of age, gender, stage, histology, large mediastinal adenopathy and B symptoms suggests that the presence of pleural effusion is a factor to consider in the determination of treatment approaches.
